# PD-L1 Expression in Muscle Invasive Urothelial Carcinomas as Assessed via Immunohistochemistry: Correlations with Specific Clinical and Pathological Features, with Emphasis on Prognosis after Radical Cystectomy

**DOI:** 10.3390/life11050404

**Published:** 2021-04-28

**Authors:** Ioan Alin Nechifor-Boilă, Andrada Loghin, Adela Nechifor-Boilă, Myriam Decaussin-Petrucci, Septimiu Voidăzan, Bogdan Călin Chibelean, Orsolya Martha, Angela Borda

**Affiliations:** 1 Department of Anatomy and Embryology, George Emil Palade University of Medicine, Pharmacy, Science and Technology of Târgu-Mureș, 540142 Târgu-Mures, Romania; ioan.nechifor-boila@umfst.ro; 2 Department of Urology, Mureș County Hospital, 540142 Târgu-Mures, Romania; orsolya.martha@umfst.ro; 3 Department of Histology, George Emil Palade University of Medicine, Pharmacy, Science and Technology of Târgu-Mureș, 540142 Târgu-Mures, Romania; andrada.loghin@umfst.ro (A.L.); adela.nechifor-boila@umfst.ro (A.N.-B.); angela.borda@umfst.ro (A.B.); 4 Department of Pathology, Mureș County Hospital, 540011 Târgu-Mureș, Romania; 5 Centre Hospitalier Lyon Sud, Department of Pathology, Universite Claude Bernard Lyon 1, 69310 Pierre-Bénite, France; myriam.decaussin-petrucci@chu-lyon.fr; 6 Department of Epidemiology, George Emil Palade University of Medicine, Pharmacy, Science and Technology of Târgu-Mureș, 540142 Târgu-Mures, Romania; septimiu.voidazan@umfst.ro; 7 Department of Pathology, Emergency Mureș County Hospital, 540136 Târgu Mureș, Romania

**Keywords:** muscle-invasive bladder cancer, tumor stage, lymphatic node status overall survival, immune checkpoint inhibitors

## Abstract

In the present study, we analyzed Programmed Death Ligand-1 (PD-L1) expression in radical cystectomy (RC) specimens from patients with muscle-invasive urothelial carcinoma (UC), in order to assess any correlations with specific clinicopathological features and its potential prognostic value. A multi-institutional study was performed within the departments of urology and pathology at the Mureș County Hospital, Romania, and Centre Hospitalier Lyon Sud, France. Sixty-nine patients with MIBC were included, for whom tumor histology (conventional versus histological variant/differentiation), tumor extension (T), lymph node involvement (N), and distant metastases (M) were recorded. PD-L1 immunostaining was performed using the 22C3 clone and was interpreted using the combined positive score (CPS) as recommended (Dako Agilent, Santa Clara, CA, USA). Positive PD-L1 immunostaining was more prevalent among UCs with squamous differentiation compared to conventional UCs and trended towards an improved OS (*p* = 0.366). We found the T stage to be a risk factor for poor survival in PD-L1-positive patients (HR 2.9, *p* = 0.021), along with the N stage in PD-L1-negative patients (HR 1.98, *p* = 0.007). No other clinicopathological factor was found to be significantly associated with PD-L1 positivity. Thus, we confirm the need for PD-L1 immunostaining prior to initiating immune checkpoint inhibitor therapy for a more accurate assessment of the patients’ chances of responding to treatment.

## 1. Introduction

Bladder cancer (BC) is the fifth most common cancer in Europe and the twelfth most frequent globally, with estimated incidence rates of 23 and 7.2 per 100,000 inhabitants in Europe and worldwide, respectively. In 2018, the mortality rate for BC in the European Union was 7.7/100,000 inhabitants [1]. Urothelial carcinomas (UCs) account for the great majority of BC cases, most being detected as non-muscle-invasive tumors. Although locally advanced and metastatic cases (≥pT2) represent only a quarter of UCs, they do account for the highest mortality rates in BC and are thus regarded as a “high-risk” subpopulation [2]. To date, few treatment options are available for these patients. Over the past 30 years, cisplatin-based chemotherapy has been the gold standard treatment despite its serious toxicity (making almost half of patients ineligible) and moderate improvement of outcome with rare complete and/or durable remissions [3].

The introduction of immune checkpoint inhibitor drugs aimed at blocking the Programmed Death-1 (PD-1)/Programmed Death Ligand-1 (PD-L1) pathway offered a new perspective. These drugs limit the interaction of PD-1 expressed on T cells with its ligand PD-L1, which is expressed by tumor cells to “escape” the immune response, thereby improving T-cell-mediated immune responses [4]. They were recently approved by the United States Food and Drug Administration (FDA) and the European Medicines Agency (EMA) for the treatment of cisplatin-ineligible or cisplatin-recurrent patients with positive PD-1/PD-L1 immunohistochemical status [5,6,7,8,9,10].

Currently, there are promising ongoing studies aimed at exploring the use of immune checkpoint inhibitor drugs in adjuvant and neoadjuvant settings. To date, positive results have been reported for nivolumab (CheckMate 274) and pembrolizumab (PURE-01), while results from other trials are pending (adjuvant pembrolizumab in the AMBASSADOR trial, NCT03244384). However, according to recent results from the DANUBE trial, durvalumab both alone and in combination therapy failed to produce a significant improvement in overall survival when compared to standard chemotherapy [11].

PD-L1 expression as determined by immunohistochemistry (IHC) was the first clinically validated predictive factor of UC treatment outcome to be translated into clinical practice, and PD-L1 is today the most extensively studied biomarker in UCs [10]. Nevertheless, data regarding its potential prognostic and/or predictive value in UCs are conflicting among reports from different trials [8,12,13,14]. The inherent morphological and genomic heterogeneity observed in UC cases may influence PD-L1-positive expression rates. Additionally, the presence of UC variants (in almost one third of cases) and their potential negative impact on survival rates could also influence PD-L1 expression [15,16].

In the present study, we used IHC to analyze PD-L1 expression in radical cystectomy (RC) specimens from patients with MIBCs who received neither adjuvant preoperative chemotherapy nor radiotherapy. Levels of expression were evaluated in terms of their correlation with specific clinicopathological features and potential prognostic value for overall survival (OS) in these patients.

## 2. Materials and Methods

A multi-institutional board-approved study was performed on a consecutive series of patients with muscle-invasive UC who received oncological follow up after radical cystectomy (RC). Patients were selected based on a review of medical records from the department of urology, Mureș County Hospital, Târgu-Mureș, Romania (between November 2011 and October 2018) and the Service d’Urologie, Centre Hospitalier Lyon Sud, Lyon, France (between January 2016 and November 2018).

All patients underwent lymphadenectomy as a standard procedure alongside RC. However, for some patients (e.g., palliative cystectomy for massive hematuria), lymphadenectomy was not performed (n = 20, 28.0%). No patient included in the study received preoperative chemotherapy and/or radiotherapy.

Demographic and pathological data were retrieved from institutional databases and repositories, and original pathological reports from the pathology departments of the Emergency County Hospital Târgu-Mureș, Romania and Centre Hospitalier Lyon Sud, Lyon, France, respectively.

The corresponding hematoxylin–eosin (HE)-stained slides for all cases included in the study were reviewed by three experienced uropathologists to reach a consensus (AB, MDP, and AL). Tumor histology, grade, and pathological stage were assigned according to the 2016 World Health Organization (WHO) Classification of Tumors of the Urinary System and Male Genital Organs [15] and the 2017 American Joint Committee on Cancer/Union for International Cancer Control (AJCC/UICC) TNM Classification of Tumors [17]. The following data were recorded: tumor histological type (conventional UC versus histological variant/differentiation of UC), association of a papillary component [18], associated carcinoma in situ (CIS), tumor extension (T stage), lymph node involvement (N stage), distant metastases (M stage), and presence of positive surgical resection margins. Only patients with muscle-invasive (≥pT2) tumors were included in the study. Lymph node metastasis was defined as involvement of at least one regional lymph node (N+). Positive distant metastases (M1) were considered if secondary and metastatic tumors were identified at the time of diagnosis or during the follow-up period.

The overall survival (OS) was defined as the time interval between the initial surgical treatment and the last available follow-up data (clinical assessment and/or life status: dead or alive). For the Romanian cohort, follow-up data were collected both from the Romanian National Insurance System database, and the Târgu-Mureș County Hospital institutional database. For the French cohort, follow-up data were available from the institutional database of the Service d’Urologie, Centre Hospitalier Lyon Sud.

All patients had regular follow-up visits scheduled according to guidelines issued by the Romanian Ministry of Health and the European Association of Urology (EAU) Guidelines on Muscle-Invasive and Metastatic Bladder Cancer [2,19]. Recurrent disease was defined as tumor recurrence in patients previously classified as disease free.

For each case, one representative formalin-fixed paraffin-embedded (FFPE) block was selected for IHC assay. PD-1/PD-L1 status was assessed immunohistochemically using the 22C3 clone (PharmDx, Agilent Technologies, Santa Clara, CA, USA). In accordance with the manufacturer’s recommendations (PD-L1 IHC 22C3 PharmDx Interpretation Manual [20]), the selected FFPE block comprised at least a 30% invasive tumor component, with no or limited necrosis and including the tumor invasion front.

PD-L1 IHC was performed using 4 µm full sections. All staining was carried out using a Ventana BenchMark Ultra automated slide stainer (Ventana, Tucson, USA), and antibody visualization was achieved using the OptiView DAB IHC Detection Kit (Ventana Medical Systems, Oro Valley, AZ, USA) according to the manufacturer’s instructions. Specimens were stained with the PD-L1 IHC clone 22C3 (Dako, Agilent Pathology Solutions, Santa Clara, CA, USA; monoclonal; retrieval CC1 64 min, incubation 32 min, ready to use dilution). Positive controls were included in every run (positive control tonsil tissue).

All slides were examined and consensus was reached by four pathologists with a special interest in uropathology, trained at scoring PD-L1 on IHC (MDP, AL, ANB, AB). The entire tumor regions on the whole-slide sections were evaluated. Positive PD-L1 staining was considered only in the cellular membrane (and cytoplasm when present) of tumor cells, as well as membrane and/or cytoplasmic staining of mononuclear inflammatory cells (MIC) involved in response to the tumor [20].

PD-L1-positive versus -negative expression status was assessed using the combined positive score (CPS), defined as the total number of PD-L1-positive stained cells, both tumor (with positive membrane staining) and immune (with membrane and/or cytoplasmic staining), divided by the total number of viable tumor cells and multiplied by 100. As recommended by Bellmunt et al. [7] and subsequently included in the PD-L1 IHC 22C3 pharmDx Interpretation Manual [20], PD-L1 staining was considered positive if CPS was ≥ 10.

Descriptive statistics (mean, median, and standard deviation) were performed for continuous variables. The Mann-Whitney test was used to assess the statistical significance of differences in medians between two independent variables with a non-Gaussian distribution. For variables with a normal distribution, Student’s *t*-test was used. Relationships between two categorical variables were assessed using the *chi*-squared test.

Survival analysis was performed using both the Kaplan-Meyer and Cox regression methods. The Kaplan-Meyer method was used to assess OS in the study group in relation both to the presence of the different variants of UC and to positive PD-L1 expression. Univariate and multivariate Cox regression were used to analyze the impact of PD-L1 expression on OS after adjusting for all available confounders. Statistical significance was considered at *p* < 0.05. Statistical analysis was performed using the SPSS Statistics 23.0 (SPSS, IBM Corp, Armonk, USA).

## 3. Results

### 3.1. Demographic and Tumor Characteristics

Our study included 69 patients who underwent RC for MIBC (Table 1). Among them, 47 patients had been operated on within the urology department of Târgu-Mureș, Romania and 22 within the Service d’Urologie, Centre Hospitalier Lyon Sud, Lyon, France.

The majority of patients were men (n = 57, 82.6%) with only 17.4% (n = 12) of the sample being women. The mean age of the patients was 67.35 ± 9.98 years.

The UCs were conventional in more than half of the cases (n = 36, 52.2%), followed by 11 (15.9%) poorly differentiated, 5 (7.2%) micropapillary, 5 (7.2%) with squamous differentiation, 4 (5.8%) sarcomatoid, 3 (4.3%) plasmacytoid, 1 (1.4%) with glandular differentiation, 1 (1.4%) nested, and 3 (4.3%) mixed. Concomitant CIS was documented in 24 (34.8%) cases.

Concerning the tumor stage, the majority of cases were pT3 (n = 34 cases, 49.3%), followed by pT4 (n = 20, 29%) and pT2 (n = 15, 21.7%). Lymph node dissection was performed in 49 (71%) cases. Of these, 24 cases (49%) displayed lymph node involvement. Distant metastases were documented in 13 (18.8%) patients (present at the time of surgery or that developed during the follow-up period).

Positive margins were found following surgical resection of 8 (11.6%) UCs: 1 conventional (pT4a), 4 poorly differentiated (2 pT3, 1 pT2 and 1 PT4), 2 plasmacytoid variant, and 1 micropapillary variant.

### 3.2. Association of PD-L1 Expression with Clinical and Tumor Features

Tumor cell staining with PD-L1 was present in any degree in 31 (44.9%) patients at a mean percentage of 19.8 ± 30.8%. Upon grading the levels of intensity from 0 to 3, the majority of positive cases were grade 3 (n = 14, 45.1%), followed by grade 2 (n = 9, 29%) and grade 1 (n = 8, 25.8%).

Immune cell staining with PD-L1 was present to some degree in 59 cases (85.5%) at a mean percentage of 16.7 ± 20.1%. Considering the same levels of intensity as for tumor cells, the majority was also grade 3 staining (n = 27, 45.7%), followed by grade 2 (n = 26, 44%) and grade 1 (n = 6, 10.1%).

When applying the combined positive score (CPS) at a threshold of 10 (as recommended by the manufacturer), a substantial proportion of UC cases (n = 28, 40.6%) displayed positive PD-L1 immunostaining.

Table 1 documents the clinical and pathological features of the study cases stratified according to PD-L1 expression status (positive versus negative). PD-L1 positivity showed no significant association with either age (*p* = 0.22) or gender (*p* = 0.33) of the patients.

**Figure 1:**
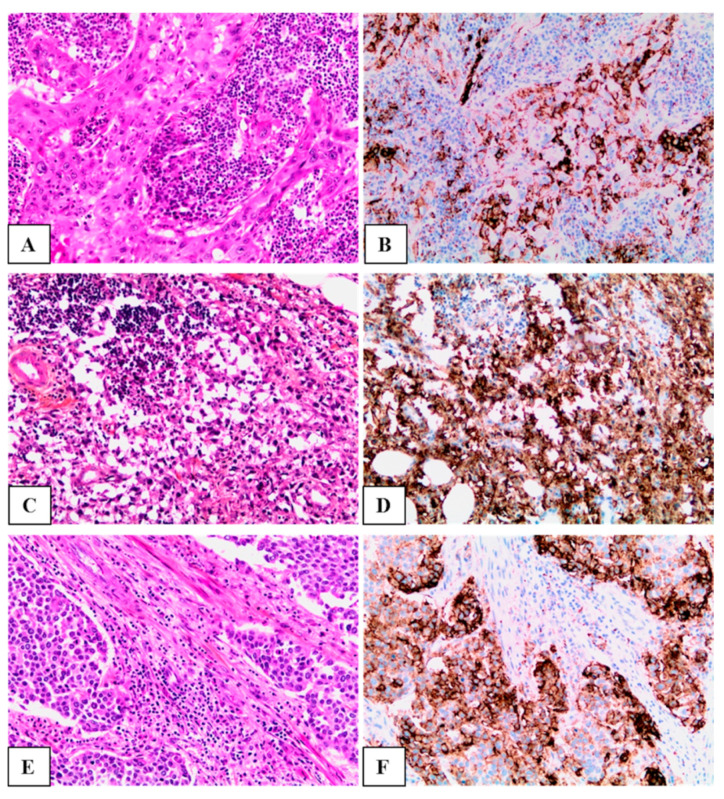
Representative examples of urothelial carcinoma cases (HE staining left) with a positive 22C3 PD-L1 status (right). (**A**,**B**) A case of urothelial carcinoma with squamous differentiation demonstrating moderate to intense, partial or complete membrane PD-L1 expression in most of the tumor cells, and a clumpy, less intense, cytoplasmic ± membrane positivity in the immune cells; the CPS for this case was set at 50. (**C**,**D**) A case of sarcomatoid variant urothelial carcinoma, revealing an intense, complete PD-L1 membrane positivity in all tumor cells; the CPS for this case was set at 100. (**E**,**F**) A case of conventional urothelial carcinoma showing complete membrane expression in the tumor cells and a clumpy, less intense, cytoplasmic ± membrane pattern of expression in the immune cells; the CPS for this case was set at 80.

Among PD-L1-positive cases, nonconventional UCs (n = 17, 60.7%) (Figure 1A–D) were significantly more frequent than conventional (n = 11, 39.3%) (Figure 1E,F).

PD-L1 positivity rates were the highest in UCs with squamous differentiation (Figure 1A,B). Among the abovementioned PD-L1-positive variant UC cases, 4 were poorly differentiated, 3 were sarcomatoid (Figure 1C,D), 2 were micropapillary, 2 were mixed, and 1 showed glandular differentiation (see Table 1).

Positive surgical resection margins showed no relationship with PD-L1 status (*p* = 0.13).

TNM stage also showed no association, with no statistically significant differences observed between PD-L1-positive and PD-L1-negative cases in terms of either tumor stage (*p* = 0.72) or lymph node involvement (*p* = 0.67).

### 3.3. Association of PD-L1 Expression with Clinical Outcome Following RC

Follow-up data were available for all the patients included in the study. None of the patients were treated with anti-PD1 or anti-PD-L1 checkpoint inhibitors. Forty patients died during the follow-up period.

Distant metastases (lung, liver, ovary, brain, adrenal gland, bone, peritoneum, or generalized) were documented in 13 (18.8%) patients, with no differences associated with PD-L1-positive status (*p* = 0.86). Histologically, 5 cases were conventional UCs and 8 cases were nonconventional UCs (1 with squamous differentiation, 1 micropapillary variant, 2 sarcomatoid variants, 1 poorly differentiated UC, and 2 plasmacytoid variants). Tumor recurrence was rare, being demonstrated in only 3 cases (4.3%).

The OS ranged from 1 to 83 months, with median and mean survival rates of 10 and 17.51 months, respectively (95% CI: 12.51–22.5).

**Figure 2:**
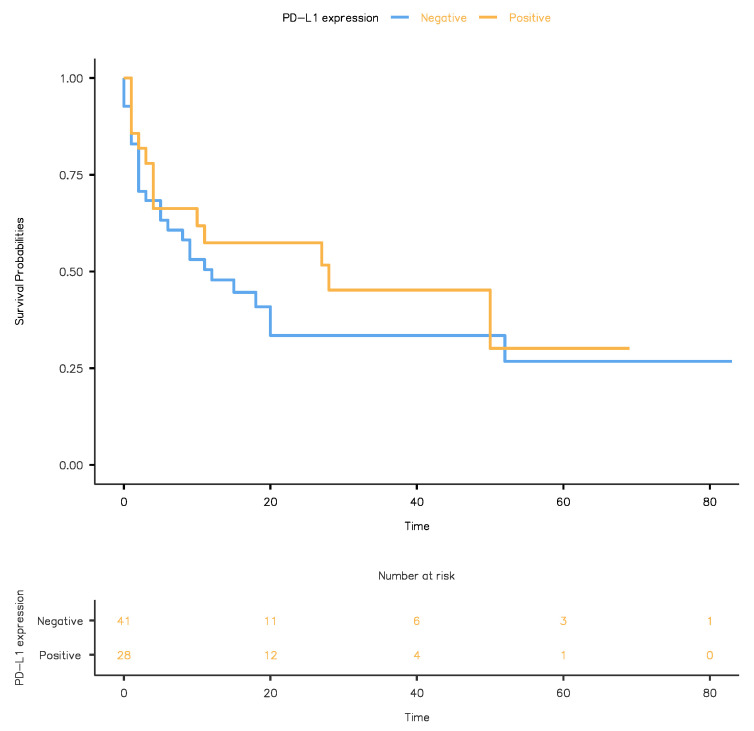
Kaplan-Meier plots for overall survival showing that patients with urothelial carcinoma and a positive PD-L1 status (orange line) had improved overall survival versus patients with urothelial carcinoma and a negative PD-L1 status (blue line).

We analyzed the association between PD-L1 status and OS using the Kaplan–Meyer and the Cox regression methods. PD-L1-positive staining was associated with improved OS compared to PD-L1-negative cases, although the difference did not reach statistical significance (*p* = 0.366): 28 months (95% CI: 0–58.6) versus 12 months (95% CI: 3.98–20) (Figure 2). This result was also supported by a separate Cox analysis that found an increased OS in PD-L1-positive cases compared to PD-L1-negative ones: HR 1.34, (95%CI: 0.697–2.58) (*p* = 0.379).

Furthermore, we evaluated the impact of various clinical and pathological factors on the OS of the patients with UC included in our study, stratified according to PD-L1 status. We found no statistically significant association between OS and gender (*p* = 0.438), concomitant CIS (*p* = 0.324), pT stage (*p* = 0.337), lymph node involvement (*p* = 0.383), distant metastases (*p* = 0.380), positive surgical resection margins (*p* = 0.497), or tumor recurrence (*p* = 0.389), regardless of PD-L1 expression (data not shown).

Next, the OS was stratified according to PD-L1 status (positive versus negative). Cox univariate analysis revealed that among the PD-L1-positive subgroup of patients, pT stage significantly influenced OS (HR 2.91, 95% CI: 1.175–7.21, *p* = 0.021) (Table 2). This result was further confirmed by multivariate analysis (HR 3.018, 95% CI: 1.132–8.046, *p* = 0.027) (Table 2 and Table 3).

Among the PD-L1-negative subgroup of patients, both univariate and multivariate Cox analyses revealed lymph node involvement as a risk factor for poor OS: *p* = 0.07, HR 1.982, (95%CI: 1.205–3.263), and *p* = 0.008, HR 2.61, 95% CI: 1.287–5.26], respectively (Table 2 and Table 3).

## 4. Discussion

In the present study, we analyzed 22C3 PD-L1 IHC expression in RC specimens from patients with MIBC not treated with adjuvant preoperative chemotherapy and/or radiotherapy in order to assess the prognostic value of PD-L1 and its potential correlation with specific clinicopathological features.

We found that PD-L1 was expressed in a substantial proportion of our MIBC patients (40.6%). Moreover, PD-L1-positive cases were significantly more prevalent within the nonconventional UC group compared to the conventional UC group (*p* = 0.048). These results are in accordance with previous published data in the literature. In their study, Pichler et al. [12] reported a substantial, intense positive PD-L1 staining among nonconventional UCs (variants or UCs with divergent differentiation) compared to conventional UCs, which were generally PD-L1 negative (46.2% vs. 20.8%; *p* = 0.002). In comparison, our study has the advantage of having analyzed a larger number of nonconventional UCs, present in almost equal proportions to the conventional UCs (n = 33/69, 47.8% in our study versus n = 13/61, 21.2% in the Pichler study).

In our study, PD-L1-positive staining was found in almost all nonconventional UCs, but mainly among UCs with squamous differentiation, which were all PD-L1 positive. In their study, Reis et al. [3] compared the expression of three PD-L1 clones (SP263, SP 242, and 22C3) in variant UC cases, and found that UCs with squamous differentiation were more frequently positive than the other variants (14 positive cases out of 16 included in the study.

Concerning the clinical factors, PD-L1 expression appeared not to be influenced by age or gender in the patients in our study. This is in accordance with the study of Holland et al., in which neither age nor gender of the patients were found to be significantly associated with PD-1/PD-L1 expression [21]. In their broad meta-analysis, Ding et al. [22] also found no significant association between PD-L1 expression and gender (*p* = 0.138).

Our data demonstrated no association between 22C3 PD-L1 IHC expression and tumor stage, lymph node involvement and distant metastases, concomitant CIS, or positive surgical resection margins. This was in accordance with the study by Xylinas et al. [23] that found no correlation between PD-L1 expression and tumor stage, grade, presence of distance metastasis, concomitant CIS, or positive soft tissue surgical margins. Similarly, Pichler et al. [12] found no statistically significant association between tumor stage or lymphatic node involvement and PD-L1 expression.

We found that PD-L1-positive staining was not a risk factor for poor OS (*p* = 0.379). By contrast, PD-L1-positive staining was associated with improved OS compared to PD-L1-negative staining in our study, although the difference did not reach statistical significance (*p* = 0.366). Similarly, while Xylinas et al. [23] found no association between PD-L1 (B7-H1) expression and disease recurrence, cancer-specific mortality, or overall mortality, they did reveal a tendency towards a positive association with increased overall mortality (HR: 1.97, CI: 0.99–3.89, *p* = 0.06) in a subcohort of organ-confined UC patients. Pichler et al. [12], on the other hand, showed that patients with UC variants had lower OS rates when compared to patients with conventional UC. Their analysis found that high-intensity PD-L1 staining had a hazard ratio (HR) of 2.25 for death, while the presence of variant UC had a HR of 2.48 for death. Furthermore, in another study, the same authors reported no difference in recurrence-free survival rate in locally advanced UC patients treated with RC when considering different PD-L1 staining levels for tumor cells. However, PD-L1 staining on immune cells was revealed to be a predictor of poorer recurrence-free survival [13].

We found that tumor stage represents a risk factor for poor OS in the subgroup of PD-L1-positive UC patients (as assessed by both univariate and multivariate Cox regression analysis). Concerning the PD-L1-negative subgroup of UC patients, lymph node involvement was found to be a risk factor for poor OS. However, both tumor stage and lymph node involvement are well-known risk factors in survival in RC patients. This is supported by the study of Madersbacher et al., who showed statistically significant decreases in OS as pT stage and pN stage increased. Thus, they reported a decrease in the 5-year OS from 63% in patients with pT1 tumors to 32% in those with pT4 tumors [24]. Marks et al. [25] demonstrated that age, tumor stage, soft tissue surgical margin, and lymph node involvement status are independent predictive factors for the OS in patients with UC treated with RC. Thus, patients with lymph node involvement and extranodal extension had an increased mortality when compared to patients with lymph node involvement alone or those with no lymph node involvement (HR 3.51, 95% CI: 2.10–5.86, *p* ≤ 0.001 and HR 2.47, 95% CI: 1.62–3.78, *p* ≤ 0.001, respectively).

The present study had several limitations, including its retrospective nature. However, this enabled us to obtain complete and long-term follow-up data. In addition, comparing the results of PD-L1 IHC testing remains difficult as several commercially available and experimental clones exist, each with their own interpretation scores and quantification methods, as well as particular technical requirements (e.g., different automatic staining machines) [26]. On the other hand, efforts are being made to standardize and many authors have demonstrated good analytical correlation among different PD-L1 clones, including Ventana SP263, 22C3 pharmDx, and 28-8 pharmaDx [3,5,27,28].

Still, immunohistochemical staining (not only in the case of PD-L1) in solid cancers can be influenced in a negative manner by various technical issues, including time length and type of tissue fixation, section preparation, and the quantity of tissue used (whole sections or tissue microarrays) presenting further challenges for reproducibility [29].

## 5. Conclusions

In our study, positive PD-L1 IHC expression was more prevalent among nonconventional UCs than conventional UCs, and especially prevalent among UCs with squamous differentiation. PD-L1-positive staining was associated with improved OS compared to PD-L1-negative status, although this tendency did not reach statistical significance. Tumor stage was found to be a risk factor for poor survival in PD-L1-positive, MIBC patients. In our series of patients, age, gender, TNM stage, and other pathological features, such as concomitant CIS and positive resection margins, were not found to be significantly associated with PD-L1 positivity.

## Figures and Tables

**Table 1:** Clinical, pathological, and follow-up data for the study cases, stratified according to programmed death 1 ligand 1 (PD-L1) expression.

Characteristic	Total (n = 69)	PD-L1 Positive (CPS ≥ 10) n = 28 (40.6%)	PD-L1 Negative (CPS < 10) n = 41 (59.4%)	*p*
Age (mean ± SD)	67.35 ± 9.98	69.11 ± 9.25	66.15 ± 10.4	0.22 ^‡^
Gender (n (%))				
Male	57 (82.6)	25 (89.3)	32 (78.0)	0.33 ^†^
Female	12 (17.4)	3 (10.7)	9 (22.0)	
Histology. n (%)				
UC conventional	36 (52.2)	11 (39.3)	25(61.0)	
UC nonconventional (variants):				
Poorly differentiated	11 (15.9)	4 (14.3)	7 (17.1)	
Micropapillary	5 (7.2)	2 (7.1)	3 (7.3)	
Squamous differentiation	5 (7.2)	5 (17.9)	0	
Glandular differentiation	1 (1.4)	1 (3.6)	0	
Plasmocitoid	3 (4.3)	0	3 (7.3)	
Sarcomatoid	4 (5.8)	3 (10.7)	2 (4.8)	
Nested	1 (1.4)	0	1 (2.4)	
Others (mixed)	3 (4.3)	2 (7.1)	1 (2.4)	
Assoc. papillary component (n (%))				
Absent	49 (71.0)	22 (78.6)	27 (65.9)	0.29 ^†^
Present	20 (29.0)	6 (21.4)	14 (34.1)	
Concomitant CIS (n (%))				
Absent	45 (65.2)	16 (57.1)	29 (70.7)	0.30 ^†^
Present	24 (34.8)	12 (42.9)	12 (29.3)	
Surgical margins status (n (%))				
Negative	61 (88.4)	27 (96.4)	34 (82.9)	0.13 ^†^
Positive	8 (11.6)	1 (3.6)	7 (17.1)	
Primary tumour (pT) (n (%))				
pT2	15 (21.7)	5 (17.9)	10 (24.4)	
pT3	34 (49.3)	15 (53.6)	19 (46.3)	0.72 ^†^
pT4	20 (29.0)	8 (28.6)	12(29.3)	
Lymph node involvement (n (%))				
Nx	20 (29.0)	7 (25.0)	13 (31.7)	
N0	25 (36.2)	12 (42.9)	13 (31.7)	0.67 ^†^
N+ (including N1, N2, N3)	24 (34.8)	9 (32.1)	15 (36.6)	
Distant metastasis (n (%))				
M0	56 (81.2)	23 (82.1)	33 (80.5)	0.86 ^†^
M1	13 (18.8)	5 (17.9)	8 (19.5)	
Follow-up data (median months)	10 (0–83)	10.5 (1–69)	9 (0–83)	0.68 ^^^
Tumor recurrence (n (%))				
Absent	66 (95.7)	27 (96.4)	39 (95.1)	0.79 ^†^
Present	3 (4.3)	1 (3.6)	2 (4.9)	
Death (n (%))				
No	29 (42.0)	14 (50.0)	15 (36.6)	0.26 ^†^
Yes	40 (58.0)	14 (50.0)	26 (63.4)	

CPS: combined positive score; ^†^ *chi* square test; ^‡^ Student *t*-test; ^^^ Mann-Whitney test; SD: standard deviation.

**Table 2:** Univariate Cox analysis of multiple clinical and pathological factors stratified according to PD-L1 expression status.

PD-L1-Positive Group (CPS ≥ 10)					PD-L1-Negative Group (CPS < 10)			
Clinical Factors	*p*-Value	Hazard Ratio (HR)	95.0% CI for HR		*p*-Value	Hazard Ratio (HR)	95.0% CI for HR	
			Lower	Upper			Lower	Upper
Gender	0.699	0.666	0.085	5.217	0.246	1.679	0.700	4.028
Age	0.924	0.997	0.940	1.058	0.280	1.020	0.984	1.057
T stage	0.021	2.910	1.175	7.210	0.439	1.239	0.720	2.131
N stage	0.599	1.189	0.625	2.261	0.007	1.982	1.205	3.263
M stage	0.347	1.752	0.544	5.647	0.227	1.710	0.717	4.082
Carcinoma in situ	0.208	1.974	0.685	5.685	0.674	1.190	0.529	2.679
Positive surgical margins	0.333	2.795	0.349	22.405	0.463	1.445	0.541	3.861
Tumor recurrence	0.624	0.046	0	10,387.347	0.443	0.455	0.061	3.400

HR: hazard ratio; CPS: combined positive score.

**Table 3:** Multivariate Cox analysis of multiple clinical and pathological factors stratified by PD-L1 expression status.

PD-L1-Positive Group					PD-L1-Negative Group			
Clinical Factors	*p*-Value	Hazard Ratio (HR)	95.0% CI for HR		*p*-Value	Hazard Ratio (HR)	95.0% CI for HR	
			Lower	Upper			Lower	Upper
Gender	0.750	0.671	0.058	7.812	0.402	1.538	0.562	4.213
Age (years)	0.481	0.973	0.903	1.049	0.193	1.029	0.986	1.074
T stage	0.027	3.018	1.132	8.046	0.659	0.870	0.469	1.613
N stage	0.441	1.375	0.612	3.091	0.008	2.601	1.287	5.260
M stage	0.547	1.527	0.385	6.052	0.098	2.625	0.837	8.234
Tumor recurrence	0.991	0	0	0	0.819	0.766	0.078	7.508
Positive surgical margins	0.898	1.174	0.100	13.745	0.977	0.981	0.269	3.572
Carcinoma in situ	0.767	1.346	0.189	9.589	0.881	1.076	0.413	2.807

## Data Availability

The datasets used and/or analyzed during the current study are available from the corresponding author on reasonable request.
